# Hypophosphatemia–factors associated with its development and 90-day mortality effect: a prospective observational study

**DOI:** 10.3389/fmed.2026.1858326

**Published:** 2026-06-29

**Authors:** Liran Statlender, Tzippy Shochat, Limor Rozilyo, Izhack Cherny, Jumana Amer, Guy Halperin, Eyal Robinson, Moran Hellerman Itzhaki, Itai Bendavid, Guy Fishman, Pierre Singer, Ilya Kagan

**Affiliations:** 1 Department of General Intensive Care, Rabin Medical Center, Beilinson Hospital, Petah Tikva, Israel; 2 School of Medicine, Tel Aviv University, Tel Aviv, Israel; 3 Statistical Consulting Unit, Rabin Medical Center, Petah Tikva, Israel; 4 Clinical Biochemistry Laboratory, Beilinson Hospital, Rabin Medical Center, Petah Tikva, Israel; 5 Clinical Endocrinology Laboratory, Beilinson Hospital, Rabin Medical Center, Petah Tikva, Israel; 6 Institute for Nutrition Research, Felsenstein Medical Research Center, Petah Tikva, Israel; 7 ICU, Dina Recanati School of Medicine, Reichman University, Herzliya, Israel

**Keywords:** creatinine clearance, hypophosphatemia, prognostication, refeeding syndrome, urine collection

## Abstract

**Background:**

Several factors are associated with hypophosphatemia. There is a paucity of data regarding the effects of restrictive energy delivery and phosphate renal handling with hypophosphatemia in the critically ill patient.

**Methods:**

This is a single-center observational study. Patients were included if they were admitted ventilated and their ICU stay was at least 48 h. Baseline characteristics were recorded upon admission; other variables were collected daily during the first five admission days: SOFA1 score, serum phosphate, energy delivery, phosphate delivery and fluid balance. A 6-h urine collection for phosphate and creatinine was performed daily. Creatinine clearance (CrCl_6hr_), tubular reabsorption of phosphate (TRP), and tubular maximal reabsorption rate of phosphate adjusted to the glomerular filtration rate (TmP/GFR) were calculated. Mortality follow up time was 90 days. A comparison was made between patients who developed hypophosphatemia (serum phosphate < 2.5 mg/dl), and those who did not. Univariate and multivariate regressions for hypophosphatemia and for 90-day mortality were performed.

**Results:**

One hundred eighty-one patients were included in the analysis. One hundred and nine (60.22%) developed hypophosphatemia. They were younger, had lower APACHE2 score, and were admitted more frequently due to trauma. No association of energy delivery with hypophosphatemia was found. In a multivariate analysis only log of CrCl_6hr_ and TmP/GFR were associated with hypophosphatemia. Hypophosphatemia was associated with 90-d mortality in univariate analysis, but not in multivariate analysis.

**Conclusion:**

Creatinine clearance based on a 6-h urine collection and TmP/GFR were associated with hypophosphatemia, whereas energy delivery was not. Age and APACHE2 were associated with 90-d mortality, but hypophosphatemia was not.

## Background

Hypophosphatemia is a common disorder in critically ill patients, appearing in 15%–35% of ICU admissions (1–3). Hypophosphatemia is generally accepted as a predictor of poor outcomes, such as prolonged ventilation (4–6) and higher mortality (7–9). However, conflicting evidence exists and several works demonstrated no effect on the length of ventilation (10, 11), nor mortality (1, 2, 10, 12). Our group demonstrated both a negative association between hypophosphatemia and mortality (13), and a lack of association between hypophosphatemia and mortality (14), when examining two retrospective cohorts.

Phosphate is mainly an intracellular anion (only 1% of its total body amount is extracellular). It is mainly absorbed in the jejunum and excreted in the urine (15). Serum phosphate level is regulated by vitamin D, parathyroid hormone (PTH), and fibroblast growth factor 23 (FGF-23) (16, 17). Three processes affect serum phosphate level: (1) phosphate shift to extracellular space from the intestine, bone, and intracellular space; (2) Glomerular filtration rate (GFR); and (3) TmP/GFR - tubular maximal reabsorption rate of phosphate (TmP) adjusted to the GFR (to avoid GFR effect on TmP) (18, 19).

### Renal effect on phosphate regulation

Phosphate is filtered in the glomerulus and reabsorbed mainly in the proximal tubule by sodium-phosphate cotransporters (15). Tubular reabsorption of phosphate (TRP) is the fraction of phosphate in the glomerular filtrate that is reabsorbed. TRP ≤ 0.86 signifies that tubular reabsorption rate is at its maximal capacity (18). TmP/GFR is the theoretical threshold of serum phosphate level, below which there would be no urine excretion of phosphate (18, 19). Both TRP and TmP/GFR are calculated based on phosphate and creatinine measurements from urine and serum samples (18, 20, 21). Calculations based on random urine samples are poorly correlated with calculations based on a 24-h urine collection (22).

The effect of kidney function in critically ill patients with hypophosphatemia has been scarcely described. The expected kidney response hypophosphatemia is increased phosphate reabsorption (23), while only a minority of cases are result of increased renal loss (24–26).

### Phosphate shift to/from the extracellular space: energy delivery effect

Phosphate shift to/from the extracellular space is affected by many factors, including phosphate balance (intestinal intake vs. phosphate excretion), calcium and phosphate-regulating hormones (PTH and vitamin D) (24), pH (27), insulin levels (28, 29) and energy delivery (30).

The term “refeeding hypophosphatemia” was coined to highlight the importance of nutritional support initiation as a cause of hypophosphatemia development (4), likely explained by cellular reuptake with energy delivery after prolonged starvation (31). Critically ill patients are considered at risk for refeeding syndrome, even without considering other risk factors (which are not considered as hypophosphatemia risk factors by themselves in the critically ill patient (32–34). Evidence suggests that early and increased energy delivery is harmful (35, 36), supporting current guidelines of slow progression to energy delivery goal (33). This recommendation currently applies to all critically ill patients suffering from prolonged starvation, as no risk stratification tool exists (32).

The primary outcome of this study, was to explore the association of both energy delivery (under a restrictive energy delivery strategy as regularly done in our ICU) and renal phosphate handling with hypophosphatemia development. As a secondary outcome, we aimed to describe the association between hypophosphatemia and 90-day mortality.

## Materials and methods

### Participants, procedure, and setting

A prospective observational study was performed in an 18-bed, mixed surgical-medical ICU in a university-affiliated tertiary hospital. The study was approved by the Institutional Review Board (RMC-23-0149) in accordance with the Helsinki Declaration and registered at clinicaltrials.gov (registration number NCT06779331). Informed consent was waived, due to the observational nature of the study. Although several laboratory analyses were performed solely for the study purposes (urine creatinine and urine phosphate levels from urine collection, and PTH measurements), neither of these required any further intervention with the patient, beyond routine, regular clinical workup.

Consecutive adult patients aged over 18 years who were ventilated and urinary catheterized on the first midnight of their admission, and whose admission lasted for more than 48 h, were included in the study. The enrollment period was from September 10th, 2023, through January 7th, 2025 (recruitment was postponed between October 7th, 2023, and January 9th, 2024, as the ICU was moved to a sheltered facility). We excluded patients with (1) chronic kidney failure who received dialysis; (2) patients who needed acute kidney replacement therapy (KRT) within 48 h of admission; (3) patients who underwent urological manipulation (including nephrostomy, suprapubic catheterization, nephrectomy, or cystectomy); (4) patients with a known phosphate metabolism disorder; (5) patients who were transferred from another critical care unit; (6) patients who were admitted for brain death evaluation; and (7) patients from whom a decision of palliative care was decided within 48 h of admission. Due to financial constraints, the study was limited to enrollment of 200 patients.

### Measures

We collected the following demographic data for each patient: age, sex, weight, body mass index (BMI), and admission category (medical, surgical, or trauma). The Acute Physiologic and Chronic Health Evaluation II (APACHE II) score was calculated on admission. Mortality follow-up was defined as 90 days.

During the first five admission days, the sequential organ failure assessment (SOFA1) score was calculated daily; Energy delivery (whether enteral or parenteral nutrition, or hidden calories), day of initiation of nutritional energy delivery (whether enteral or parenteral) and phosphate amount delivered to each patient were recorded daily from the EMR (in our EMR software, each nutritional component and glucose or lipid containing solution, is assigned its energy content (in terms of kcal/ml; As the rate of these treatments is recorded, hourly and daily summation of energy delivery is recorded). Energy delivery was adjusted to the patient’s weight. Daily energy delivery goal was defined as 25 kcal/kg of patient actual weight (37, 38). The percentage of daily energy delivery from the daily energy delivery goal was calculated as (daily energy delivered/daily energy delivery goal) × 100. Daily fluid balance and the amount of insulin given were recorded.

Serum creatinine and phosphate were measured upon admission, daily at 06:00 am as part of routine blood testing, and upon treating physician’s decision. A 6-h urine collection was performed from midnight to 06:00 am, starting from the first midnight after admission, during the first 5 nights of admission. Urine collection volume, urine creatinine (Ucr) and urine phosphate (Upi) levels at the collection, and total urine output were recorded. Based on the urine collection, several calculations were performed daily, including:


$$-\mathrm{Creatinine}\mathrm{clearance}\left({\mathrm{CrCl}}_{6\,h\,r}\right)=\left(\mathrm{Ucr}\times \mathrm{CollectionVolume}\right)/\left(\mathrm{Scr}\times 360\right);$$

-TRP=1-((Upi*Scr)/(Spi*Ucr))⁢(18)

-TmP/GFR=(18):

°ifTRP>0.86:TmP/GFR=Spi*TRP

$${}^{\circ}\mathrm{if}\mathrm{TRP}\leq 0.86^{\prime \prime }\mathrm{Tmp}/\mathrm{GFR}=\mathrm{Spi}*(\left(0.3*\mathrm{TRP}\right)/\left(1-0.8*\mathrm{TRP}\right)).$$

Patients were considered to have Hypophosphatemia if the phosphate level was less than 2.5 mg/dL in any chemistry test taken from admission until completion of the last urine collection.

Daily phosphate balance was estimated by subtracting the total amount of phosphate excreted in the urine collection (multiplied by 4) from the amount of delivered daily phosphate.

Parathyroid hormone (PTH) levels, and vitamin D levels were measured (using the routine chemistry blood analysis withdrawn from each patient) once during the first five admission days. Due to financial restrictions, it was measured only for the last 50 patients enrolled.

### Statistical analysis

Continuous variables are presented as medians (IQR); Categorical variables are presented as counts and percentages. A *p*-value of less than 0.05 was considered significant in all analyses.

Based on the variables’ distribution, we performed several transformations: Log of CrCl_6hr_; Log of total energy delivery; Log of weight-adjusted energy delivery; Log of percentage of daily energy delivery goal. Daily fluid balance is presented in centiliters (cl); Daily phosphate balance is presented in centigrams (cg).

Baseline characteristics and 90-day mortality were compared between patients who developed hypophosphatemia during the first five admission days and those who did not, using Mann-Whitney or Chi-square tests as appropriate. Due to the anticipated large proportion of trauma patients at the time of the study period, an interaction was examined: between age and admission reason regarding hypophosphatemia development; between hypophosphatemia development and admission reason regarding mortality. We also performed an analysis excluding trauma patients.

### Hypophosphatemia development

We used univariate time-varying Cox proportional hazards models to describe the association of baseline characteristics and daily changing variables [SOFA1, log of daily energy delivery (total-, weight-adjusted- and percentage of energy goal), log of CrCl_6hr_, TRP, TmP/GFR, phosphate balance, and fluid balance] with hypophosphatemia development. Covariate values were updated during follow-up to reflect changes in exposure status, and risk estimates were recalculated for each interval. This approach accounts for dynamic factors that may influence the hazard of the outcome over time. A multivariate analysis for hypophosphatemia development was performed, including baseline variables, and significant covariates from the univariate analysis. If several significant covariates were correlated [whether by a high correlation coefficient, or conceptually (i.e., APACHE2 and SOFA1)], only one of them entered the regression. PTH was also not used in the multivariate analysis as it was measured only for 50 patients.

### 90 -day mortality evaluation

To assess factors associated with 90-day mortality, we used univariate Cox proportional hazards regression. For this analysis, we used an extreme value of each patient from the five surveillance days: minimal log CrCl_6hr_; minimal TmP/GFR, minimal TRP, maximal log energy delivered (total-, weight-adjusted, and percentage of caloric goal), and minimal phosphate balance. To assess the association of hypophosphatemia with 90-day mortality, we also performed multivariate analysis including baseline characteristics and hypophosphatemia.

We excluded patients whose hypophosphatemia was evident before completion of the first urine collection from the analyses; as a patient was considered to have hypophosphatemia had it occurred during the first 5 admission days, we excluded patients who died (without developing hypophosphatemia) within these days from these analyses.

Data were collected and managed using Research Electronic Data Capture (REDCap) tools (a secure, web-based software platform designed to support data capture for research studies) hosted at Clalit Health Services (39, 40).

Statistical analysis was performed using SAS v9.0 (Cary, NC, USA).

## Results

During the study period, 824 patient admissions to the ICU were screened. Two hundred patients were recruited for the study. Fourteen patients had hypophosphatemia before completion of the first urine collection; Five patients died within 5 days of admission, without hypophosphatemia; Thus, 181 patients were included in the analysis. A flowchart of study design is presented in Supplementary Figure 1.

**FIGURE 1:**
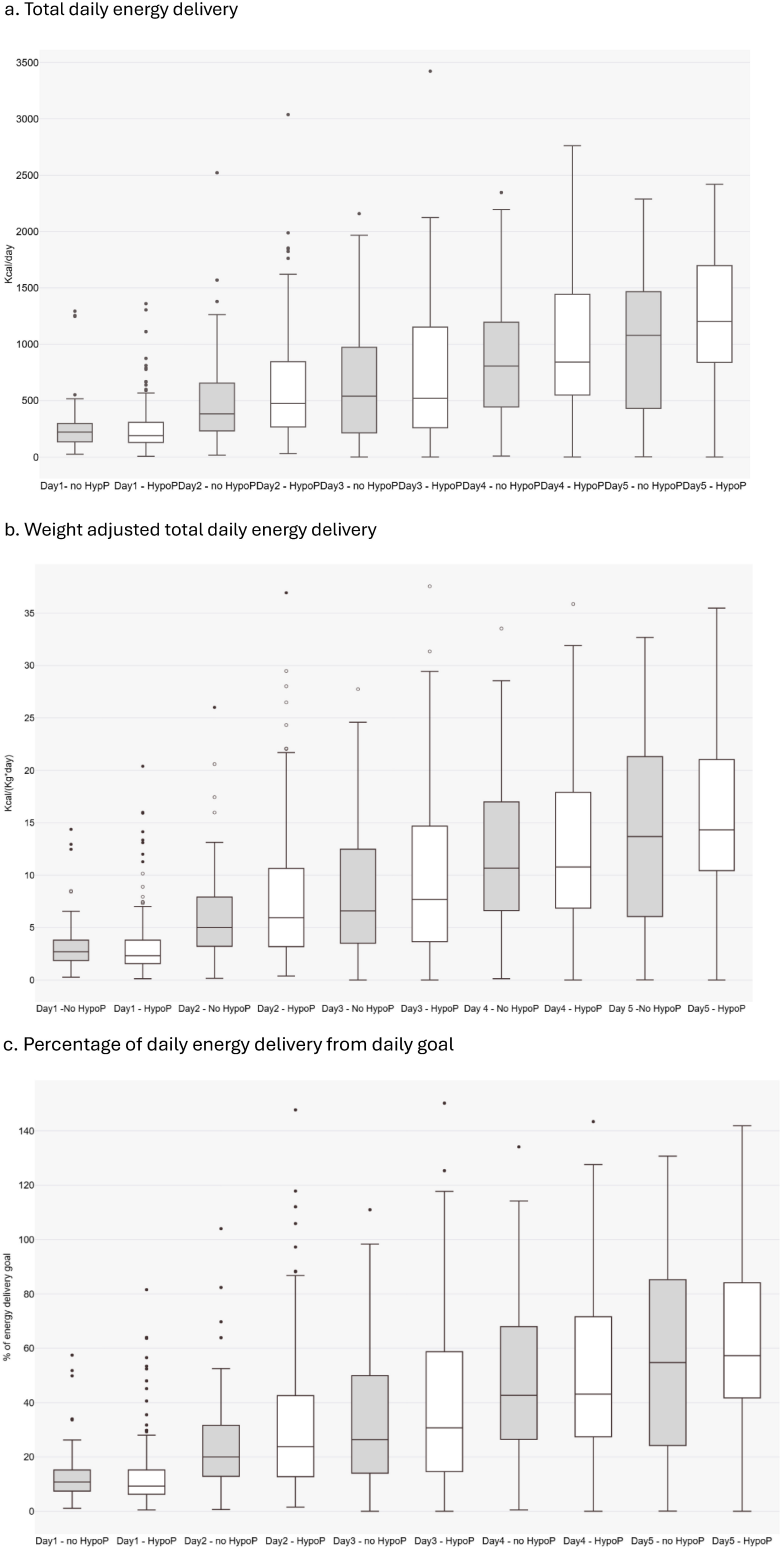
Daily energy delivery box plots by hypophosphatemia status. **(a)** Total daily energy delivery. **(b)** Weight adjusted total daily energy delivery. **(c)** Percentage of daily energy delivery from daily goal.

One hundred and nine (60.22%) patients developed hypophosphatemia within the first five admission days. Forty-four (24.31%) patients on the 1st admission day, 31 (17.13%) patients on the 2nd; Eighteen (9.94%) patients on the 3rd, 11 (6.07%) patients on the 4th, and 5 (2.76%) on the 5th. A comparison of daily CrCl_6hr_ and daily number of patients who developed hypophosphatemia by admission reason is presented in Supplementary Table 1. A summary of daily energy delivery is presented in Figure 1. Detailed daily energy delivery by route of admission (and numbers of patients who developed hypophosphatemia) is presented in Supplementary Table 2.

Fifty (27.62%) patients died within 90 days of ICU admission. A significant difference was noted in 90-day mortality between patients who developed hypophosphatemia and those who did not [22/109, (20.18%) vs. 28/72 (38.88%), *p* = 0.0058].

### Hypophosphatemia development

A comparison of baseline characteristics is presented in Table 1. Patients who developed hypophosphatemia were significantly younger (52.44 vs. 64.42, *p* = 0.0003), had lower APACHE2 score (18 vs. 22, *p* < 0.0001), and were admitted more frequently due to trauma (42.2%, vs. 18.06%, *p* = 0.025). An interaction between age and admission reason was not found (coefficient − 0.691, *p* = 0.708). Results of univariate time-varying Cox regression for hypophosphatemia development are presented in Table 2. Besides the aforementioned differences in baseline characteristics, the log of CrCl_6hr_ (HR 1.93 95% CI 1.65–2.26, *p* < 0.0001) was found to be significantly associated with hypophosphatemia development; The following variables were found to be negatively associated with hypophosphatemia development: daily SOFA1 score (HR 0.91, 95% CI 0.86–0.95, *p* < 0.0001), PTH levels (HR 0.94, 95% CI 0.89–0.99, *p* = 0.018), and TmP/GFR (HR 0.43 95% CI 0.37–0.50, *p* < 0.0001). None of the energy delivery variables examined, energy delivery initiation, vitamin D, phosphate balance, TRP, fluid balance, or insulin administered, were found to be significantly associated with hypophosphatemia development.

**TABLE 1:** Baseline characteristics of patients by hypophosphatemia development.

Parameter	Without hypophosphatemia	With hypophosphatemia	*P*-value
*N*	72 (39.78%)	109 (60.22%)	
Age (years)	64.42 (51.33, 72.68)	52.44 (34.28, 70.50)	0.0003
Male sex	55 (76.39%)	79 (72.48%)	0.9966
BMI (kg/m^2^)	26.39 (23.50, 30.49)	26.12 (23.15, 29.38)	0.619
Admission reason			
Medical	25 (34.72%)	36 (33.03%)	Ref
Surgical	34 (47.22%)	27 (24.77%)	0.0752
Trauma	13 (18.06%)	46 (42.20%)	0.0247
APACHE2	22 (17, 26)	18 (14, 22)	<0.0001

Categorical variables are presented as number (percentage); continuous variables are presented as medians (IQR); BMI, body mass index; APACHE2, acute physiology and chronic health evaluation 2 score.

**TABLE 2:** Univariate time-varying COX PH regression for hypophosphatemia development.

Parameter	Hazard ratio	95% lower confidence limit for hazard ratio	95% upper confidence limit for hazard ratio	*P*-value
Baseline characteristics				
Age	0.984	0.976	0.993	0.0003
Male sex	1.001	0.688	1.456	0.9966
BMI	0.992	0.961	1.024	0.6190
Admission reason (Ref = medical)				
Surgical	0.661	0.419	1.043	0.0752
Trauma	1.537	1.056	2.238	0.0247
APACHE2 score at admission	0.954	0.933	0.975	<0.0001
Daily changing variables				
SOFA1 score	0.907	0.864	0.951	<0.0001
Log of CrCl_6hr_	1.927	1.647	2.255	<0.0001
Daily fluid balance (cL)	0.999	0.985	1.013	0.8892
Energy delivery initiation (enteral or parenteral)	1.403	0.926	2.126	0.1104
Energy delivery initiation (enteral only)	1.296	0.866	1.940	0.2075
Energy delivery initiation (parenteral only)	1.263	0.624	2.557	0.5160
Phosphate metabolism				
PTH (pg/ml)	0.936	0.886	0.989	0.0179
Vitamin D (nmol/L)	1.074	0.982	1.175	0.1185
Log of insulin given (IU)	0.980	0.925	1.038	0.4864
Daily phosphate balance (centigrams)	0.986	0.966	1.007	0.1889
TRP	1.143	0.458	2.849	0.7742
TmP/GFR	0.427	0.368	0.496	<0.0001
Energy delivery				
Log of daily energy delivery (kcal/d)	1.082	0.858	1.366	0.5049
Log of daily weight-adjusted energy delivery (kcal/Kg*d)	1.096	0.868	1.384	0.4426
Log of percentage of daily energy delivery goal	1.068	0.846	1.349	0.5793

BMI, body mass index; APACHE2, acute physiology and chronic health evaluation 2 score; SOFA1, sequential organ failure assessment score (ver1); CrCl_6hr_, creatinine clearance calculation based on 6-h urine collection; PTH, parathyroid hormone; TmP/GFR, tubular maximum of phosphate, adjusted to glomerular filtration rate.

Multivariate analysis for hypophosphatemia development is presented in Table 3, using baseline characteristics, and the significantly associated covariates (excluding SOFA1 and PTH) demonstrated that only log of CrCl_6hr_ (HR 1.62, *p* = 0.0008), and TmP/GFR (HR 0.44, *p* < 0.0001) were significantly associated with hypophosphatemia development.

**TABLE 3:** Multivariate Cox PH regression for hypophosphatemia development.

Parameter	Hazard ratio	95% hazard ratio confidence limits		*P*-value
Age	1.000	0.988	1.011	0.9616
Male sex	0.713	0.450	1.129	0.1495
BMI	0.829	0.522	1.319	0.4311
Admission reason (Ref = medical)				
Surgical	0.896	0.521	1.542	0.6918
Trauma	0.961	0.586	1.576	0.8742
APACHE2	0.989	0.956	1.022	0.4972
Log of CrCl_6hr_	1.615	1.222	2.135	0.0008
TmP/GFR	0.441	0.360	0.540	<0.0001

BMI, body mass index; APACHE2, acute physiology and chronic health evaluation 2 score; CrCl_6hr_, creatinine clearance calculation based on 6-h urine collection; TmP/GFR, tubular maximum of phosphate, adjusted to glomerular filtration rate.

### Mortality analysis

A comparison of 90-day surviving patients with non-surviving patients is presented in Supplementary Table 3. Surviving patients were significantly younger (52.11 vs. 70.31, *p* < 0.0001), more frequently of male sex (78.63% vs. 62%, *p* = 0.015), and admitted more frequently due to trauma (41.22% vs. 10%, *p* = 0.0089). Their APACHE2 score was lower (18 vs. 22.5, *p* < 0.0001); maximal SOFA score during first five admission days was lower (9 vs. 11, *p* < 0.0001); minimal CrCl_6hr_ (and log of CrCl_6hr_) was higher (58.09 ml/min vs. 23.16 ml/min, *p* < 0.0001); and cumulative fluid balance was lower (0.17 cl vs. 14.36 cl, *p* = 0.0017). An interaction between admission reason and hypophosphatemia development was not found (coefficient for surgical admission reason −0.617, *p* = 0.325; for trauma admission reason − 15.296, *p* = 0.988).

Significant differences were also noted regarding phosphate metabolism. Surviving patients had hypophosphatemia more frequently [87/131 (66.41%) vs. 22/50 (44%), *p* = 0.006]. Their PTH levels were lower (38 ng/ml vs. 70 ng/ml, *p* = 0.002). Their minimal TRP was higher (0.63 vs. 0.52, *p* = 0.06), and their minimal phosphate balance was more negative (−1268.32 mg/d vs. −694.12 mg/d, *p* = 0.002). No significant difference was noted regarding the minimal TmP/GFR (1.69 mg/dl vs. 1.73 mg/dl, *p* = 0.428), vitamin D levels (34.2 IU/l, vs. 27.6 IU/l, *p* = 0.082), nor any of the energy delivery variables examined.

Multivariate analysis for 90-day mortality is presented in Table 4. Only age (HR 1.02, 95% CI 1.00–1.04, *p* = 0.018) and APACHE2 (HR 1.08, 95% CI 1.03–1.12, *p* = 0.0006) were significantly associated with mortality [a trend of trauma admission reason with decreased mortality was noted (HR 0.4, 95% CI 0.14, 1.1, *p* = 0.077)]. Hypophosphatemia was not significantly associated with mortality. A multivariate analysis for 90-day mortality excluding trauma patients is presented in Supplementary Table 4. There were no differences in this analysis, except that age was not found to be significantly associated with mortality.

**TABLE 4:** Multivariate COX PH regression for 90-day mortality.

Parameter	Hazard ratio	95% hazard ratio confidence limits		*P*-value
Age	1.024	1.004	1.044	0.0180
Male sex	0.627	0.342	1.152	0.1325
BMI	0.972	0.923	1.024	0.2919
Admission reason (Ref = medical)				
Surgical	1.307	0.687	2.485	0.4150
Trauma	0.398	0.143	1.103	0.0765
APACHE2	1.077	1.032	1.123	0.0006
Hypophosphatemia	0.801	0.441	1.456	0.4665

## Discussion

In this single-center observational study hypophosphatemia prevalence was about 60%, slightly higher than previously reported (1–3). This is likely due to the high proportion of trauma patients in our cohort (32.59%), a group in which higher rates of hypophosphatemia were reported (41).

Several factors were significantly associated with increased rates of hypophosphatemia (Trauma admission reason, Log of CrCl_6hr_), while others were associated with a decreased rate (age, APACHE2, daily SOFA1 score, PTH levels and daily TmP/GFR). In a multivariate analysis, only log of CrCl_6hr_ and TmP/GFR were found to be associated with hypophosphatemia development. Neither daily phosphate balance, nor energy delivery (whether total-, weight- adjusted-, or percentage of caloric goal) were associated with hypophosphatemia.

The 90-day mortality rate of 27.62% was not different from the 90-day mortality rate in our ICU during the study period (data not shown). Some variables were associated with increased mortality in a univariate analysis (age, APACHE2, SOFA1 score, PTH, daily phosphate balance), while others were associated with decreased mortality (male sex, trauma admission reason, log of minimal CrCl_6hr_, minimal TRP). Hypophosphatemia was also found to be associated with a reduced 90-day mortality rate. However, in a multivariate analysis, only age and APACHE2 were found to be significantly associated with mortality.

### Hypophosphatemia development

Hypophosphatemia etiology is multifactorial, including (but not limited to) sepsis (7), refeeding syndrome (4), insulin therapy (28), and pH changes (27). Previous studies identified some characteristics associated with hypophosphatemia development, but the results are somewhat conflicting. Younger age (10, 14), female sex (1), lower BMI (13), and a medical admission reason (1, 8, 11) were reported to be associated with a higher risk of hypophosphatemia. APACHE2 was found to be associated with either increased (1, 8) or decreased (13, 14) hypophosphatemia rates. Of note, several other studies could not demonstrate some of the aforementioned associations (4, 7, 42). Although some of these variables were found in the current study to be associated with hypophosphatemia (younger age, female sex, lower APACHE2) in the univariate analysis, none were associated with hypophosphatemia in the multivariate analysis.

Patients admitted due to trauma are usually younger, have lower mortality and are known to be prone to augmented renal clearance (43). This association between these factors might explain why they are individually associated with hypophosphatemia in univariate analysis but aren’t individual risk factors when checked together in multivariate analysis (Indeed, we could not demonstrate an interaction between age, admission reason and hypophosphatemia). Yang et al. demonstrated lower TRP during the first admission day of hypophosphatemia critically ill trauma patients (compared to non-hypophosphatemia patients) (41). This is somewhat contradictory to our results, as TRP was not associated with hypophosphatemia development. When examining the actual daily mean CrCl_6hr_ by admission reason there is a perceivable difference in favor of those admitted for trauma (Supplementary Table 1).

Serum phosphate level is determined by phosphate shifts to/from the extracellular space (including intestinal absorption), and by urinary phosphate excretion [regulated by GFR and TmP/GFR (44, 45)]. The associations we showed of CrCl_6hr_ with hypophosphatemia development, and the inverse association of TmP/GFR with hypophosphatemia (both after adjustments), are in line with current literature. It must be noted that TmP/GFR is calculated by multiplication of TRP with serum phosphate, so its association with hypophosphatemia is obvious, and a causal relationship cannot be established. Moreover, TRP was not associated with hypophosphatemia development, emphasizing the “mathematical” association of hypophosphatemia with TmP/GFR, rather than an etiological one. Indeed, renal phosphate regulation was not significantly different between thermally injured patients who developed hypophosphatemia, and multiple trauma patients who did not (46).

Our results do not demonstrate any association of energy delivery (whether as total amount delivered, weight-adjusted, or percentage of caloric goal) with hypophosphatemia, as previously shown (47). Moreover, we did not demonstrate association of hypophosphatemia development with nutritional support initiation (enteral and/or parenteral). This might seem contradictory to current literature, as energy delivery is considered a risk factor for refeeding hypophosphatemia (4, 33, 34). However, restrictive energy delivery (as performed in our ICU; Figure 1), without early parenteral supplementation to achieve caloric goals, is considered safe (35, 36), as evident from our results. It is possible that hypophosphatemia observed in earlier reports was the result of an excessive energy delivery, which was “too fast and too much.”

Among other factors relevant to phosphate shifts between intracellular and extracellular fluid, neither phosphate balance nor amount of insulin delivered was associated with hypophosphatemia. Insulin causes a shift of phosphate into the cells, and may cause hypophosphatemia (47), but this is not mandatory. Negative phosphate balance is a rare cause of hypophosphatemia (48). As previously described (24), PTH was found to be inversely associated with hypophosphatemia development. PTH has a phosphaturic effect, which is balanced by an efflux of phosphate from the bone. As we measured PTH only in 50 patients, we did not assess it in the multivariate analysis (otherwise variables would not be equally distributed among patients included).

To summarize, the only associated factors for hypophosphatemia development were CrCl_6hr_ and TmP/GFR. Those are well-described associations, reflecting a physiological response. Normal kidney function is necessary for phosphate excretion; TmP/GFR is expected to decrease with hypophosphatemia. None of the baseline variables, phosphate balance, insulin therapy, TRP, nutritional support initiation or energy delivery amount were found to be associated with hypophosphatemia. This suggests that in patients with normal kidney function. hypophosphatemia might serve as an independent marker of a patient’s status. Further studies are needed to define hypophosphatemia etiology and implications.

### Hypophosphatemia and mortality

The association of hypophosphatemia and mortality is debatable, as all possible associations were reported, whether increased mortality (7, 11), decreased mortality (8, 13, 14), or lack of association (1, 10, 12, 49). This difference is probably explained by the different underlying illness and hypophosphatemia mechanism (30). Hypophosphatemia was associated with decreased 90-day mortality only in the univariate analysis, but not after adjustment for baseline variables, among which age and APACHE2 were significantly associated with mortality.

As both age and APACHE2 were found to be associated with hypophosphatemia in the univariate analysis, it is reasonable to conclude that the univariate association of hypophosphatemia with 90-day mortality is explained by the association of age and APACHE2 with mortality. The lack of association of age and APACHE2 with hypophosphatemia in the multivariate analysis is explained by kidney function; when kidney injury ensues, to some degree hypophosphatemia is not likely. Thus, it seems that hypophosphatemia is an independent finding, which is associated with younger patients with a lower APACHE2 score and a functioning kidney. The secondary analysis of the EPaNIC RCT suggested hypophosphatemia was a marker of worse prognosis for patients randomized to early parenteral nutrition (50). This is likely explained by the increased energy delivery these patients received, and not by the hypophosphatemia *per se*. Our study suggests that under a restrictive energy delivery protocol, hypophosphatemia might indicate a better prognosis, but this is probably related to the patient’s baseline characteristics.

### Strengths and limitations

Our study has several limitations. First, generalization might be difficult due to the single-center nature of the study, and a possible selection bias, due to the inclusion of ventilated patients who survived at least 48 h. Indeed, the 200-patient enrollment limitation, of which 181 were included in the final analysis reflects about a fifth of ICU admissions during the study period. Second, urinary collection was partial (a quarter of a day) and thus might not reflect the actual kidney function. Urinary creatinine clearance which is calculated based on 24 h urine collection is a reliable estimation of GFR, although it overestimate it (51). Its 24 h nature makes it cumbersome to perform. Shorter duration urine collection is easier to perform; Creatinine clearance calculation, which is based on such a urine collection, are considered reliable GFR estimation (52–54), with some controversy (55). We used a 6-h urine collection, as it can be easily done during a nightshift by a single nurse treating the patient, despite it might miss some of the dynamics of a critically ill patient. The same is applicable to phosphate balance calculation. Third, we did not measure REE, but rather calculated the caloric goal based on a formula whose inaccuracy is well known. Fourth, due to the observational nature of the study, we did not compare different energy delivery strategies; such a comparison might allow more solid conclusions. Fifth, we did not examine whether there were differences between enteral or parenteral nutritional support; we did not examine whether there was an effect of hidden calories delivered.

On the other hand, this study has some strengths. First, its prospective nature avoids the disadvantages of retrospective studies. Second, the patient population (ventilated patients who were admitted for at least 48 h in a mixed ICU) reflects the mainstay of critical care routine work. Third, this is one of the largest series examining renal handling of phosphate during critical illness; kidney importance in phosphate balance cannot be underestimated. Fourth, the robustness of data from the complete 5 days of follow-up during ICU admission.

## Conclusion

This prospective observational study demonstrated that under restrictive energy delivery, CrCl_6hr_ and TmP/GFR were associated with hypophosphatemia, whereas energy delivery was not. Age and APACHE2 were associated with 90-d mortality, but hypophosphatemia was not.

## Data Availability

The raw data supporting the conclusions of this article will be made available upon reasonable request and after Clalit Health Services authorization.
