# Supplementary material for: Hypophosphatemia–factors associated with its development and 90-day mortality effect: a prospective observational study
Source: Front Med (Lausanne). 2026 Jun 29;13:1858326. doi: 10.3389/fmed.2026.1858326 (PMC13357668; doi:10.3389/fmed.2026.1858326)
Supplement: Supplementary file 1 [file Data_Sheet_1.docx]

Supplement Figure 1. Flowchart of study design

824 admissions during study period

355 admissions

297 admissions

247 admissions

244 admissions

223 patients

200 patients

181 patients included in final analysis

469 admissions of either not ventilated patients or LOS < 48hr

41 admissions with KRT

17 admissions with urological manipulations

25 transferred from another ICU

25 admissions of brain death evaluation or palliative therapy within 48hr from admission

1 patient is less than 18 years old

2 patients with phosphate metabolism disorder

21 readmissions

23 screening failure / missed more than two urinary collections

14 patients developed hypophosphatemia before completion of the first urinary collection

5 patients died within 5 days of admission

LOS - length of stay; KRT – kidney replacement therapy;

Supplement Table 1. Daily CrCl_6hr_ and number of patients with new hypophosphatemia by admission reason

|  |  | Day1 | Day2 | Day3 | Day4 | Day5 |
| --- | --- | --- | --- | --- | --- | --- |
| Medical | CrCl (ml/min) | 56.23  (23.9, 103.9) | 86.1  (36.36, 132.9) | 85.01  (41.76, 128.79) | 79.93  (52.19, 151.72) | 75.95  (37.7, 133.46) |
|  | hypoP patients (n) | 16 | 7 | 9 | 1 | 3 |
| Surgical | CrCl (ml/min) | 29  (10.92, 60.05) | 42.53  (9.81, 80.96) | 46.93  (16.52, 86.02) | 45.09  (20, 78.61) | 49.66  (14.66, 73.79) |
|  | hypoP patients (n) | 8 | 10 | 3 | 4 | 2 |
| Trauma | CrCl (ml/min) | 93.1  (53.57, 121.2) | 127.79  (96.43, 166.2) | 144.53  (65.92, 194.83) | 128.38  (79.39, 169.66) | 132.29  (79.81, 178.86) |
|  | hypoP patients (n) | 20 | 14 | 6 | 6 | 0 |

mean (IQR) are presented.

CrCl_6hr_ – Creatinine clearance calculation based on 6-hour urine collection

Supplement Table 2. Daily energy delivery by administration route.

2a. Number of patients and number of patients who developed hypophosphatemia by daily type of nutritional support

|  | No nutrition | Enteral nutrition | Paraenteral nutrition | Enteral & Parenteral nutrition |
| --- | --- | --- | --- | --- |
| Day1 | 159 (87.85%) | 21 (11.60%) | 1 (0.55%) | 0 |
| HypoP patients | 34 (18.78%) | 10 (5.52%) | 0 | 0 |
| Day2 | 91 (66.42%) | 40 (29.20%) | 6 (4.38%) | 0 |
| HypoP patients | 18 (13.14%) | 10 (7.3%) | 3 (2.19%) | 0 |
| Day3 | 49 (50.00%) | 42 (42.86%) | 7 (7.14%) | 0 |
| HypoP patients | 8 (8.16%) | 9 (9.18%) | 1 (1.02%) | 0 |
| Day4 | 19 (26.76%) | 40 (56.34%) | 12 (16.90%) | 0 |
| HypoP patients | 4 (5.63%) | 5 (7.04%) | 2 (2.82%) | 0 |
| Day5 | 8 (15.38%) | 33 (63.46%) | 10 (19.23%) | 1 (1.92%) |
| HypoP patients | 1 (1.92%) | 3 (5.77%) | 1 (1.92%) | 0 |

hypoP - hypophosphatemia

Patients who developed hypophosphatemia or discharged are excluded each day.

All Percentages are of daily sum of patients.

2b. Amount of energy delivery (Kcal) by route of administration according to admission days.

|  | enteral | | parenteral | |
| --- | --- | --- | --- | --- |
|  | Mean±std | Median (IQR) | Mean±std | Median (IQR) |
| Day1 | 53.98±178.3 | 0 (0, 0) | 216.44±132.28 | 181 (130, 272) |
| Day2 | 184.93±369.31 | 0 (0, 242) | 333.42±194.79 | 326 (192, 440) |
| Day3 | 306.42±449.71 | 0 (0, 571) | 317.68±271.06 | 289.5 (136, 437) |
| Day4 | 498.06±549.04 | 398 (0, 753) | 405.35±391.69 | 327 (161, 540) |
| Day5 | 690.98±646.47 | 703.5 (0, 1298.5) | 438.42±431.69 | 329 (112, 514) |

Parenteral route includes hidden calories.

Supplement Table 3. Comparison of 90-day surviving and non-surviving patients.

|  | 90-day survivors | 90-day deceased | HR for mortality | P value |
| --- | --- | --- | --- | --- |
| N | 131 | 50 |  |  |
| Age (years) | 52.11 (36.36, 69.87) | 70.31 (56.86, 74.58) | 1.04 (1.02, 1.06) | <0.0001 |
| Male Sex | 103 (78.63%) | 31 (62.00%) | 0.49 (0.28, 0.87) | 0.015 |
| BMI (kg/m^2^) | 26.17 (23.53, 30.86) | 26.62 (22.64, 29.30) | 0.97 (0.92, 1.02) | 0.259 |
| Admission Reason |  |  |  |  |
| Medical | 43 (32.89%) | 18 (36.00%) | Ref | N/A |
| Surgical | 34 (25.95%) | 27 (54.00%) | 1.57 (0.86, 2.85) | 0.141 |
| Trauma | 54 (41.22%) | 5 (10.00%) | 0.27 (0.1, 0.71) | 0.008 |
| APACHE2 | 18 (13, 22) | 22.5 (20, 28) | 1.11 (1.07, 1.15) | <0.0001 |
| Maximal SOFA1 score | 9 (8, 11) | 11 (10, 13) | 1.22 (1.11, 1.34) | <0.0001 |
| Log of minimal CrCl_6hr_ | 4.06 (3.25, 4.59) | 3.14 (1.77, 3.92) | 0.61 (0.51, 0.73) | <0.0001 |
| Sum of 5 days fluid balance (cl) | 0.17 (-18.74, 18.46) | 14.36 (-5.07, 31.18) | 1 .012 (1.005, 1.020) | 0.0017 |
| Phosphate Metabolism |  |  |  |  |
| HypoP | 87 (66.41%) | 22 (44.00%) | 0.46 (0.26, 0.8) | 0.006 |
| PTH (ng/ml) (n=50) | 38 (25, 44) | 70 (43, 105) | 1.03 (1.01, 1.04) | 0.002 |
| Vitamin d (IU/ml) (n=50) | 34.20 (25.6, 51.1) | 27.6 (17.4, 43.6) | 0.97 (0.93, 1.01) | 0.087 |
| Minimal daily phosphate balance (mg/day) | -1268.32 (-2102.40, -568.93) | -694.12 (-1107.51, -355.85) | 1.07 (1.02, 1.11) | 0.002 |
| Minimal TRP (%) | 0.63 (0.50, 0.75) | 0.52 (0.38, 0.67) | 0.17 (0.05, 0.60) | 0.006 |
| Minimal TmP/GFR (mg/dl) | 1.69 (1.09, 2.28) | 1.73 (1, 2.55) | 1.11 (0.86, 1.42) | 0.428 |
| Energy delivery |  |  |  |  |
| Log of Maximal daily energy delivery (Kcal/day) | 7.14 (6.45, 7.44) | 6.92 (6.5, 7.29) | 0.92 (0.64, 1.33) | 0.646 |
| Log of Maximal weight adjusted energy delivery | 2.68 (2.12, 3.10) | 2.66 (2.26, 2.93) | 1.06 (0.72, 1.57) | 0.752 |
| Log of Maximal percentage of energy goal | -0.54 (-1.09, -0.12) | -0.56 (-0.96, -0.29) | 1.064 (0.72, 1.57) | 0.752 |

Categorial variables are presented as number (percentage); continuous variables are presented as mean (IQR);

BMI – body mass index; APACHE2 – Acute physiology and chronic health evaluation 2 score; SOFA1 – Sequential organ failure assessment score (ver1); CrCl_6hr_ – Creatinine clearance calculation based on 6-hour urine collection; hypoP – hypophosphatemia; PTH – parathyroid hormone; TRP – Tubular reabsorption of phosphate; TmP/GFR - Tubular maximum of phosphate, adjusted to glomerular filtration rate.

Supplement Table 4. Multivariate COX PH regression for 90-day mortality without trauma patients

| **Parameter** | **Hazard Ratio** | **95% Hazard Ratio Confidence Limits** | | **P value** |
| --- | --- | --- | --- | --- |
| Age | 1.015 | 0.995 | 1.036 | 0.1348 |
| Male Sex | 0.582 | 0.309 | 1.097 | 0.0942 |
| BMI | 0.975 | 0.925 | 1.029 | 0.3574 |
| Surgical Admission reason (vs. medical) | 1.304 | 0.687 | 2.476 | 0.4173 |
| APACHE2 | 1.076 | 1.031 | 1.122 | 0.0008 |
| Hypophosphatemia | 0.640 | 0.338 | 1.213 | 0.1714 |
